# Mass Spectrometry Based-Proteomic Analysis of *Anisakis* spp.: A Preliminary Study towards a New Diagnostic Tool

**DOI:** 10.3390/genes11060693

**Published:** 2020-06-24

**Authors:** Valeria Marzano, Stefania Pane, Gianluca Foglietta, Stefano Levi Mortera, Pamela Vernocchi, Andrea Onetti Muda, Lorenza Putignani

**Affiliations:** 1 Unit of Human Microbiome, Bambino Gesù Children’s Hospital IRCCS, Piazza Sant’Onofrio 4, 00165 Rome, Italy; valeria.marzano@opbg.net (V.M.); stefano.levimortera@opbg.net (S.L.M.); pamela.vernocchi@opbg.net (P.V.); 2 Unit of Parasitology, Bambino Gesù Children’s Hospital IRCCS, Piazza Sant’Onofrio 4, 00165 Rome, Italy; stefania.pane@opbg.net (S.P.); gianluca.foglietta@opbg.net (G.F.); 3 Department of Laboratories, Bambino Gesù Children’s Hospital IRCCS, Piazza Sant’Onofrio 4, 00165 Rome, Italy; andrea.onettimuda@opbg.net; 4 Units of Parasitology and Human Microbiome, Bambino Gesù Children’s Hospital IRCCS, Piazza Sant’Onofrio 4, 00165 Rome, Italy

**Keywords:** anisakiasis, *Anisakis* spp., proteomics, MALDI–TOF MS, LC–ESI–MS/MS

## Abstract

Anisakiasis is nowadays a well-known infection, mainly caused by the accidental ingestion of *Anisakis* larvae, following the consumption of raw or undercooked fishes and cephalopods. Due to the similarity of symptoms with those of common gastrointestinal disorders, this infection is often underestimated, and the need for new specific diagnostic tools is becoming crucial. Given the remarkable impact that MALDI–TOF MS biotyping had in the last decade in clinical routine practice for the recognition of bacterial and fungi strains, a similar scenario could be foreseen for the identification of parasites, such as nematodes. In this work, a MALDI–TOF MS profiling of *Anisakis* proteome was pursued with a view to constructing a first spectral library for the diagnosis of *Anisakis* infections. At the same time, a shotgun proteomics approach by LC–ESI–MS/MS was performed on the two main fractions obtained from protein extraction, to evaluate the protein species enriched by the protocol. A set of MALDI–TOF MS signals associated with proteins originating in the ribosomal fraction of the nematode extract was selected as a potential diagnostic tool for the identification of *Anisakis* spp.

## 1. Introduction

Among food biological hazards, parasites are particularly dangerous for human health. Globalization has markedly increased the change in eating habits, including the widespread consumption of raw, marinated, or smoked fish. Moreover, a quota of food allergies of unknown origin among the general population may be due to sensitization to *Anisakis* spp. (roundworms), representing a public health issue, whose clinical manifestations are characterized by digestive disorders, asthma, dermatitis, and even anaphylaxis. Nematodes of the Anisakidae family are fish parasites that can be found all over the world. The larvae live in the gut, visceral peritoneum, and flesh of many marine fish and cephalopod species and can colonize through different trophic bridges, ensuring and widening the parasite life cycle.

Humans can become accidental hosts of the *Anisakis* parasite by eating parasitized raw or undercooked fish containing larvae in stage 3 [1,2]. Within hours of being ingested, *Anisakis* larvae penetrate the mucosal layers of the gastrointestinal tract, causing direct tissue damage that may lead to the zoonotic disease known as anisakiasis. This acute gastrointestinal form of Anisakis infection is usually transient, with the worm dying within a few weeks. It is manifested by clinical symptoms ranging from nausea to vomiting, diarrhea, mild to severe abdominal pain, and intestinal obstruction [3], mimicking other common gastrointestinal disturbances, such as acute appendicitis, gastric ulcer, or tumors, thus making the diagnosis of anisakiasis extremely difficult. Diagnosis is generally obtained through anamnestic data, endoscopy, radiography, serum-specific anti-*Anisakis* IgE determination, or surgery if the worm has embedded. Visualization and morphological identification of the larva(e), removed by endoscopy or surgery or expelled by patients’ cough is the conclusive assessment. To date, no unambiguous diagnostic criteria and laboratory algorithm have been established; the lack of highly skilled microscopists in biomedical laboratories and the high number of false positives due to cross-reactivities with numerous panallergens have underlined the need to improve the diagnostic approaches. Indeed, because of technical difficulties in their diagnosis, these infections are likely to be under-diagnosed.

Nowadays, mass spectrometry (MS) biotyping is a rapid, easy, and validated method for accurate microbial phenotypic identification (ID). It takes advantage of the matrix-assisted laser desorption/ionization time-of-flight MS (MALDI–TOF MS) technology. In particular, three platforms, i.e., MALDI Biotyper (Bruker Daltonics GmbH, Bremen, Germany), VITEK MS (bioMerieux, Marcy-l’Étoile, France), and Andromas MS (Paris, France) are currently approved for routine use and adopted by many clinical microbiological laboratories [4,5,6,7], facilitating the clinical identification of many pathogenic microorganisms, including bacteria, yeasts, and filamentous fungi. However, an increase of applications of this technology has been observed in the last 10 years, with encouraging results also in studies regarding parasites [8,9]. A recent application of MALDI–TOF MS for *Dirofilaria* and *Ascaris* protein-based profiling showed a promising scenario towards clinical applications involving nematodes biotyping [10].

The aim of this study is to extend the MALDI–TOF MS Biotyper approach to *Anisakis* spp. ID and to define a preliminary assessment of its potential in anisakiasis diagnosis. In parallel, a shotgun identification profiling of *Anisakis* proteins by liquid chromatography–electrospray ionization–tandem MS (LC–ESI–MS/MS) was performed on the two main fractions of the extraction protocol, with the aim of detecting a relationship with the specifically detected MALDI–TOF MS Biotyper signals.

## 2. Materials and Methods

Infection by several live parasite larvae isolated from salmon portions, bought in a local supermarket in Rome in late 2019, was assessed by macroscopic observation. Additionally, microscopic analysis was performed using an Axiovert 25 microscope (Zeiss, Jena, Germany). Specimens were identified as *Anisakis* spp. larvae.

### 2.1. Protein Extraction

Five specimens were washed several times in 0.9% NaCl solution, and each larva was stored in a sterile 0.9% NaCl solution in a ratio of 1:3 (parasite weight/volume of the isotonic solution) at −80 °C. Mechanical homogenization of the frozen material was carried out with a steel pestle, followed by an ultrasonic treatment of the biomass at 100% power (BactoSonic, BANDELIN electronic GmbH & Co KG, Berlin, Germany) in five cycles of 30 s. Two-hundred µL of Lysis buffer from the MALDI Sepsityper Kit (Bruker Daltonics GmbH, Bremen, Germany) was added to the resulting sample and mixed by vortexing for 10 s. After centrifugation, the pellet was suspended with 1 mL of Washing Buffer (MALDI Sepsityper Kit) and, after another centrifugation, the supernatant (“sample A”) was used for LC–ESI–MS/MS analysis.

Last, the obtained pellet was resuspended in 1.2 mL of a solution water/ethanol 1:3, and 2/3 of it was centrifuged, obtaining a second pellet (“B”), which was resuspended in 35% formic acid (FA), 15% water, and 50% acetonitrile (ACN) for MALDI–TOF MS analysis (“sample B_M_”, M as the abbreviation for MALDI–TOF MS).

**Figure 1:**
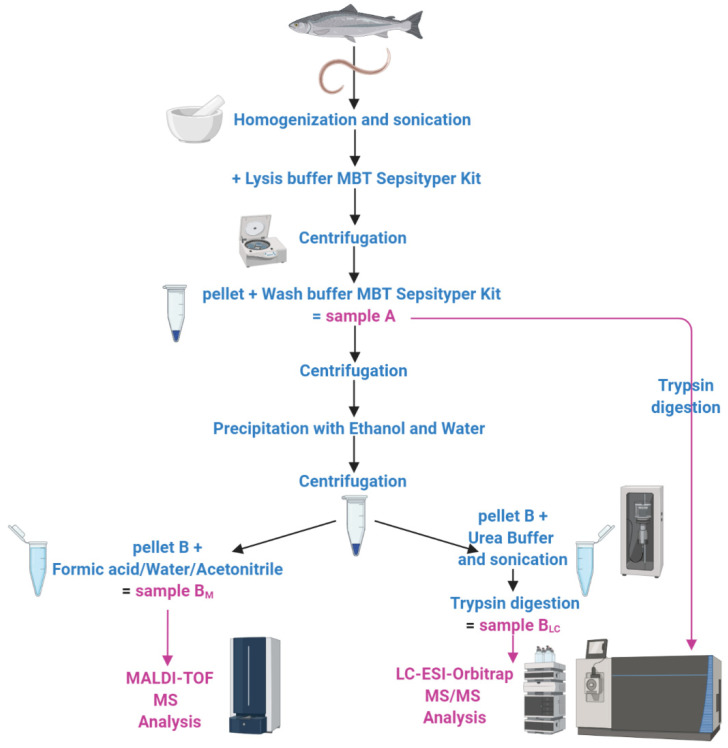
Sketch of the two proteomic experimental approaches exploited for *Anisakis* spp. identification.

In parallel, 1/3 of the solution was centrifuged for 2 min at 14,000 rpm, and the resulting pellet (“B”) was sonicated (VibraCell Ultrasonic Liquid Processor, Sonics & Materials Inc, Newtown, CT, USA) 7 times, 60% amplitude, in Sample Buffer (7 M urea, 2 M thiourea, 4% 3-[(3-cholamidopropyl)dimethylammonio]-1-propanesulfonate hydrate (CHAPS), 40 mM trizma, and 50 mM dithiothreitol (DTT)) and incubated for 1 h at 37 °C. After centrifugation at 14,000 rpm for 15 min, the supernatant (“sample B_LC_ “, LC as the abbreviation for LC–ESI–MS/MS) was collected in order to perform the LC–ESI–MS/MS analysis (Figure 1).

### 2.2. Matrix-Assisted Laser Desorption/Ionization–Time of Flight Mass Spectrometry (MALDI–TOF MS)

One microliter of sample B_M_ was placed on an MSP 96 polished steel target (Bruker Daltonics, GmbH, Bremen, Germany), air-dried, and overlaid with 1 µL of the matrix, consisting in a solution of 5 mg/mL of α-cyano-4-hydroxycinnamic acid (Bruker Daltonics GmbH, Bremen, Germany) in 50% ACN, 47.5% water, 2.5% trifluoroacetic acid (TFA). Each sample was spotted onto eight target spots of the MALDI target plate, and spectral measurements were performed with a Microflex LT mass spectrometer (Biotyper, Bruker Daltonics GmbH, Bremen, Germany), equipped with the FlexControl software package, (version 3.4, Bruker Daltonics GmbH, Bremen, Germany), operating in the positive linear mode (laser frequency 20 Hz; ion source 1 voltage, 20 kV; ion source 2 voltage, 18.4 kV; lens voltage, 6 kV; mass range, 2000 to 20,000 *m/z*). Three independent mass spectra with 240 shots (from different positions of the target spot) for each spectrum were acquired from each spot, to obtain 24 spectra replicas, externally calibrated by using the Bacterial Test Standard (Bruker Daltonics GmbH, Bremen, Germany). Subsequently, spectra datasets were imported into ClinPro Tools software (version 3.0, Bruker Daltonics, GmbH, Bremen, Germany) for data mining after peak picking on the calculated total average spectrum, setting the signal-to-noise (S/N) threshold at 3, baseline subtraction (Top Hat), and peak intensity calculation.

### 2.3. In-Solution Protein Digestion

The protein extracts (sample A and B_LC_) were subjected to reduction, alkylation, and trypsin digestion according to the filter-aided sample preparation (FASP) protocol [11]. Briefly, the protein extracts were loaded on the Microcon-10kDa Centrifugal Filter Unit with an Ultracel-10 membrane (Merck, Burlington, MA, USA) in the presence of 8 M urea and 100 mM Tris-HCl, pH 8.5; disulfide bonds were reduced for 15 min at 37 °C with 8 mM DTT, then the samples were incubated with 50 mM iodoacetamide for 15 min and subsequently with DTT and digested with 1 μg of sequencing-grade trypsin (Promega, Milan, Italy) at 37 °C in 50 mM ammonium bicarbonate buffer pH 8.0, overnight (16 h). Peptides were eluted from the Microcon, speedvac-dried, and resuspended in a water solution with 2% ACN and 0.1% FA. Total peptide content was determined by NanoDrop 2000 (Thermo Fisher Scientific, Waltham, MA, USA) analysis, with a standard curve of MassPrep *Escherichia coli* digestion (Waters, Milford, MA, USA).

### 2.4. Liquid Chromatography–Electrospray Ionization–Tandem MS (LC–ESI–MS/MS)

LC–ESI–MS/MS experiments were performed on an UltiMate3000 RSLCnano System directly coupled to an Orbitrap Fusion Tribrid mass spectrometer, operating in positive ionization mode, equipped with a nanoESI source (EASY-Spray NG) (Thermo Fisher Scientific, Waltham, MA, USA). The digested proteins (1.25 μg) were first trapped and desalted onto a μ-precolumn cartridge C18 PepMap100 (5 µm particle size, 100 Å pore size, 300 µm i.d. × 5 mm length, Thermo Fisher Scientific, Waltham, MA, USA) for 3 min at 10 μL/min, with an aqueous solution of 2% ACN and 0.1% TFA, and then separated by reverse-phase chromatography performed on an EASY-Spray PepMap RSLC C18 column (2 μm particle size, 100 Å pore size, 75 μm i.d. × 50 cm length, Thermo Fisher Scientific, Waltham, MA, USA) at a flow rate of 250 nL/min and a temperature of 35 °C, by a one-step linear gradient from 95% eluent A (0.1% FA in water) to 25% eluent B (99.9% ACN, 0.1% FA) in 113 min and total LC run of 160 min. Precursor (MS1) survey scans were recorded in the Orbitrap, at resolving powers of 120 K (at *m/z* 200). Data-dependent MS/MS (MS2) analysis was performed in top speed mode with a 3 s cycle time, during which most abundant multiple-charged (2+–7+) precursor ions detected within the range of 375–1500 *m/z* were selected for activation in order of abundance and detected in ion trap at rapid scan rate. Quadrupole isolation with a 1.6 *m/z* isolation window was used, and dynamic exclusion was enabled for 60 s after a single scan. Automatic gain control targets were 4.0 × 10^5^ for MS1 and 2.0 × 10^3^ for MS2, with 50 and 300 ms maximum injection times, respectively. For MS2, the signal intensity threshold was 5.0 ×10^3^, and the option “Injection Ions for All Available Parallelizable Time” was set. High-energy collisional dissociation (HCD) was performed using 30% normalized collision energy. Lock mass was set as an internal calibration using polydimethylcyclosiloxane (445.12003 *m/z*).

### 2.5. Database Searching and Protein Identification

Protein IDs were obtained with the embedded search engine (Sequest HT) of the Proteome Discoverer software (PD, version 2.4, Thermo Fisher Scientific, Waltham, MA, USA) after searching a custom-made database containing the complete UniProtKB/Swiss-Prot sequence entries catalogue (561,568 proteins, release: 2019_11) to which *Anisakis* UniProtKB/TrEMBL (25,874 proteins, release: 2019_11) and “Salmon” UniProtKB/TrEMBL (233,298 proteins, release: 2020_01) sequence entries were appended. The search parameters included trypsin as the proteolytic enzyme with a maximum of 2 missed cleavages per peptide allowed and oxidation of methionine as a variable modification, whereas carbamidomethylation of cysteine was set as static modification. Precursor and fragment mass tolerance were set to 10 ppm and 0.6 Da, respectively. False discovery rate (FDR) was calculated by the Percolator algorithm, and a cut-off of 0.01 was used for the identifications (i.e., the expected fraction of incorrect protein match in the entire data set was set to less than 1%, calculated on a decoy database). At least two peptides were considered for protein ID.

### 2.6. Functional Analysis

The mapping of orthologous genes from protein lists to *Caenorhabditis elegans* was carried out by the g:Orth tool of the g:Profiler web server based on data collected into the Ensembl database [12,13] and by STRING (version 11.0, database 11_0) [14,15]. Bioinformatic analyses were performed by the g:GOSt function of the g:Profiler software in order to perform statistical enrichment analysis to find over-representation of information from Gene Ontology (GO) terms and Kyoto Encyclopedia of Genes and Genomes (KEGG) biological pathways. The most significant categories associated with the uploaded datasets were identified by calculating the related significance (*p*-value) when comparing the protein list to the whole *C. elegans* proteome. The *p*-value measures the likelihood that the association between the genes/proteins in the datasets and each GO and KEGG terms is not due to random chance alone, identifying a significant over-representation of molecules in association with a given process. We applied an experiment-wide *p*-value threshold of 0.05, limiting the FDR (i.e., the expected fraction of false positives among significant terms) to less than 5%; g:GOSt uses multiple testing correction and applies the tailor-made algorithm g:SCS for reducing significance scores.

Protein–protein interaction networks analysis was performed using the STRING application. The highest confidence of 0.9 was chosen as the minimum required interaction score threshold, such that only interactions above this score were included in the predicted networks. Networks were clustered by the Markov clustering (MCL) algorithm with inflation parameter set as 3 (indirectly related to the precision of the clustering, i.e., the higher the inflation, the more abundant the clusters).

## 3. Results

### 3.1. Experimental Pipeline

After mechanical homogenization and sonication, the samples obtained from five larvae were treated according with the MALDI Sepsityper Kit protocol (Bruker Daltonics, GmbH, Bremen, Germany). To exploit the advantages of different proteomic strategies, a combined approach based on two mass spectrometry platforms was undertaken in the current study (Figure 1).

### 3.2. Protein Profiling by MALDI–TOF MS Analysis

**Figure 2:**
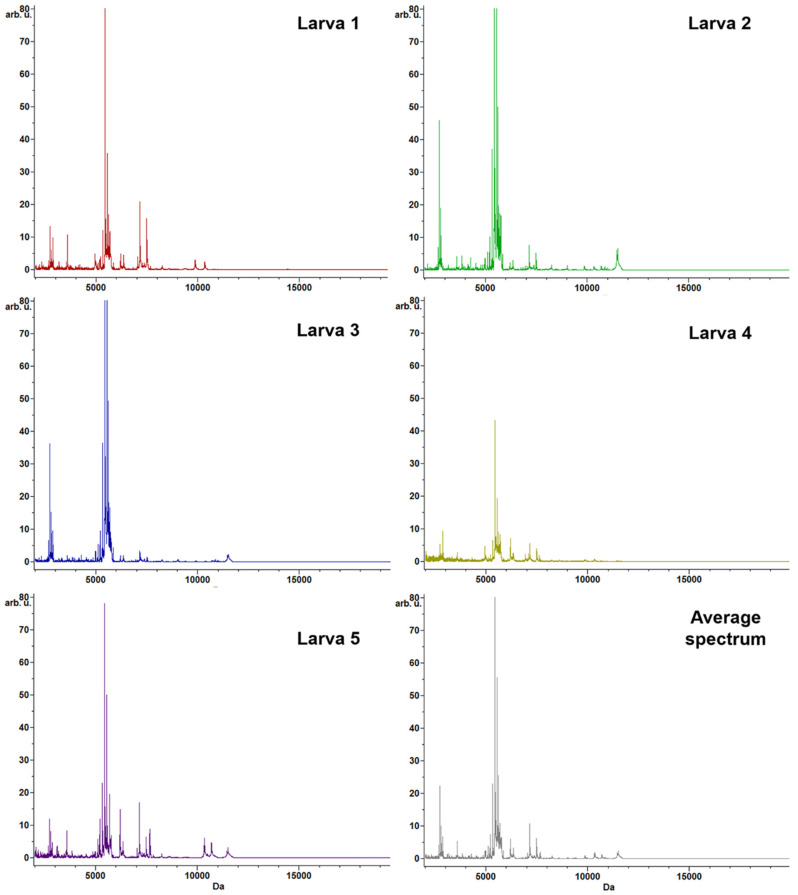
Representative protein profiling of *Anisakis* spp. by Biotyper MALDI–TOF MS. The average spectrum for each sample is depicted, as well as the average spectrum obtained from the entire spectra dataset. The *m/z* values are expressed in Da, and the signal intensities are reported in a scale of arbitrary units (arb. u.).

**Figure 3:**
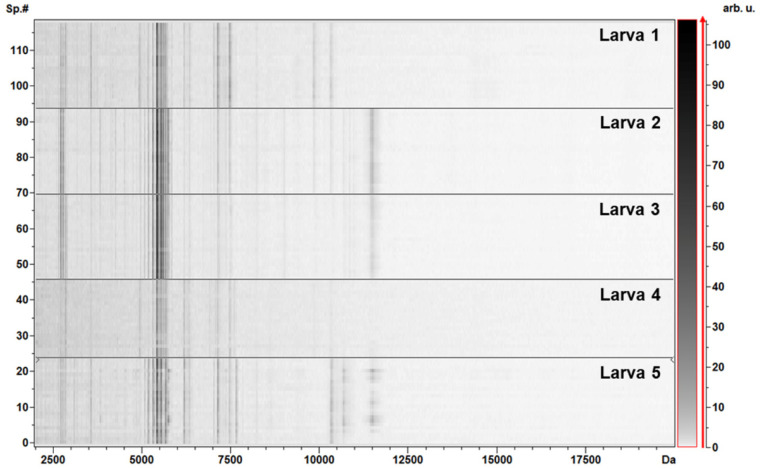
Pseudogel view of the five larvae (samples B_M_) from MALDI–TOF MS profiling. The mass-to-charge values (*m/z*, Da) are reported on the x-axis, while the gray scale bar, reported on the right y-axis, shows the relationship between the color intensity and the peak intensity, expressed by arbitrary units (arb. u.). The left y-axis evidences the spectra numbers (Sp.#).

After protein extraction, spotting, and MS analysis, high-intensity peaks in the range of 2000–12,000 Da (*m/z*) were highlighted from the five larvae (sample B_M_), with the highest density in the region comprised between 2500 and 8000 Da, with clusters of signals in ranges corresponding to 2700–2900, 5400–5700, and 7100–7500 Da (Figure 2; Figure 3).

**Figure 4:**
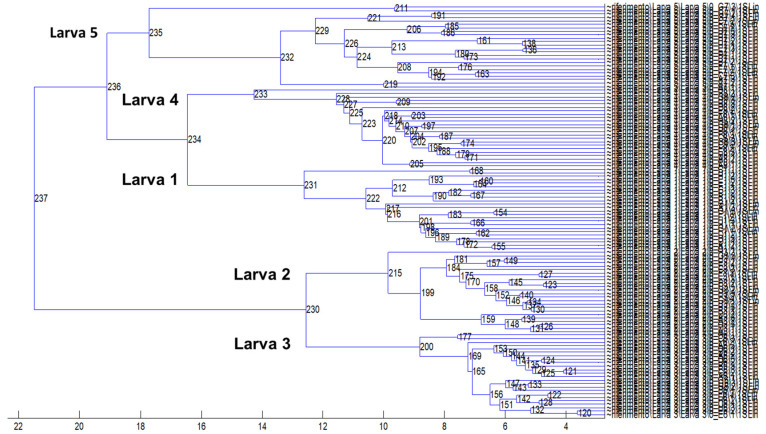
Spectra dendrogram (by unsupervised hierarchical clustering) obtained by analyzing all MALDI–TOF MS spectral replicas from *Anisakis* larvae.

The phenotypic variability of the larvae, inspected by the clustering analysis performed on the complete dataset, was underlined: mass spectra of larva 1 and 4 clustered together and formed one clade with larva 5; larva 2 and 3 were more similar and identified a different cluster (Figure 4).

The mass spectra dataset of each larva were grouped in a different class, and a cut-off ≤8 on the difference between the maximum and the minimum average peak intensities of all classes (DAve), a minimum peak intensity average of 2.5 (Ave), and 20% of the coefficient of variation (CV) for each class were applied. Regardless of the variability addressed by dendrogram analysis, 19 signals (among a total of 179) for all samples were furthermore identified, representing a collection of fingerprinting classifiers of *Anisakis* spp. (Table 1).

### 3.3. Protein Profiling by Tandem Mass Spectrometry

**Figure 5:**
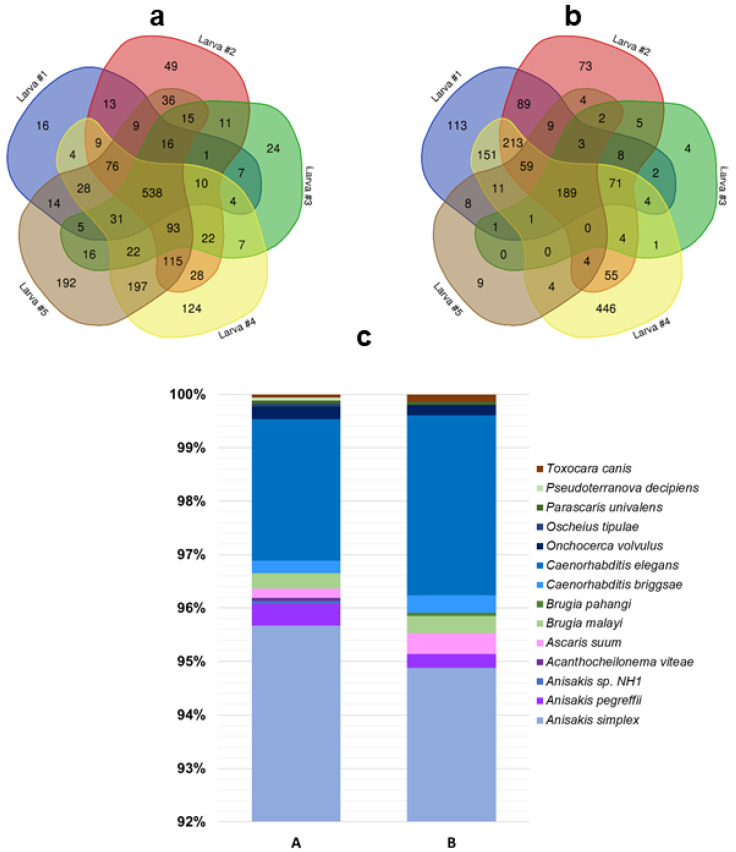
Overview of proteins identified by LC–ESI–MS/MS analysis. Venn diagrams show the number of Rhabditida proteins and their distribution in the five analyzed larvae, highlighting shared and exclusive proteins (numbers in colored areas) in samples A (**a**) and B_LC_ (**b**). The organism distribution (as percentage of identified proteins) at the Rhabditida order taxonomy level in sample A and B is depicted with histograms (**c**).

After enzymatic digestion and peptide purification, we identified the total protein content of two extraction steps of the adopted protocol by LC–ESI–MS/MS on a high-resolution platform. From sample A of larvae 1, 2, 3, 4, and 5 we identified an overall number of 2179 different proteins, of which 561 were identified as shared; in sample B_LC_, we identified a total of 3091 diverse proteins, of which 210 were common to the five larvae (Supplementary material S1). We focused our functional analysis only on identified proteins belonging to *Anisakis* species and to organisms selected according to phylogenetic similarity (Rhabditida order [16], 1732 and 1543 total different proteins in sample A and B, respectively) (Figure 5, Supplementary material S2).

**Figure 6:**
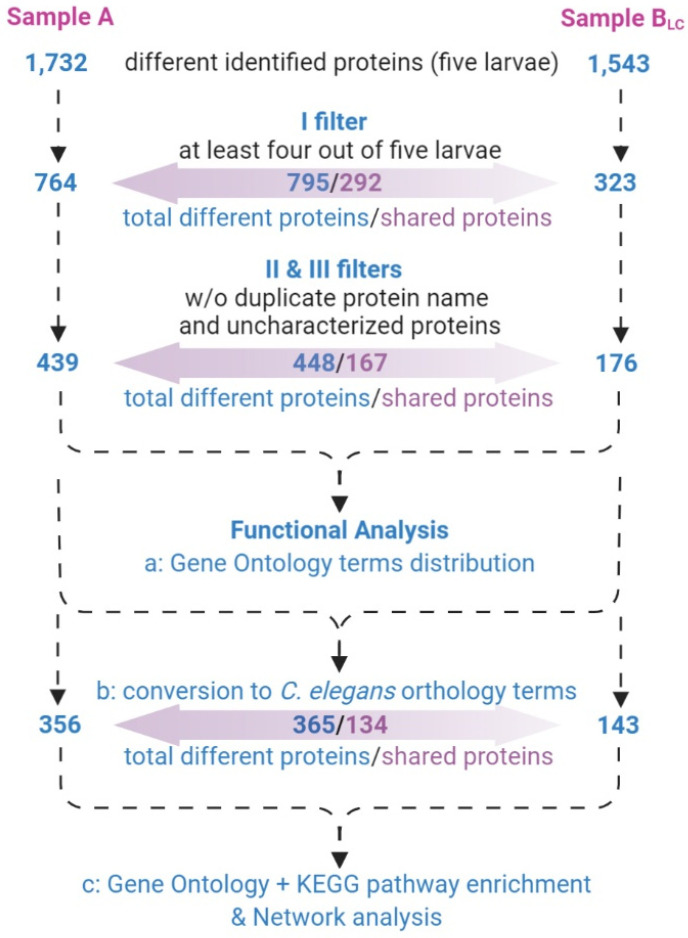
Filters applied to protein lists prior to performing functional bioinformatic analyses. KEGG, Kyoto Encyclopedia of Genes and Genomes.

Lists of identified proteins belonging to the Rhabditida order were filtered by a three-step process (Figure 6):

1. only proteins identified in at least four out of five larvae (Supplementary material S3) were considered;
2. proteins with different UniProtKB accession code but the same name were collapsed into one single hit;
3. proteins defined as unknown were deleted (Supplementary material S4).

#### Functional Analysis

**Figure 7:**
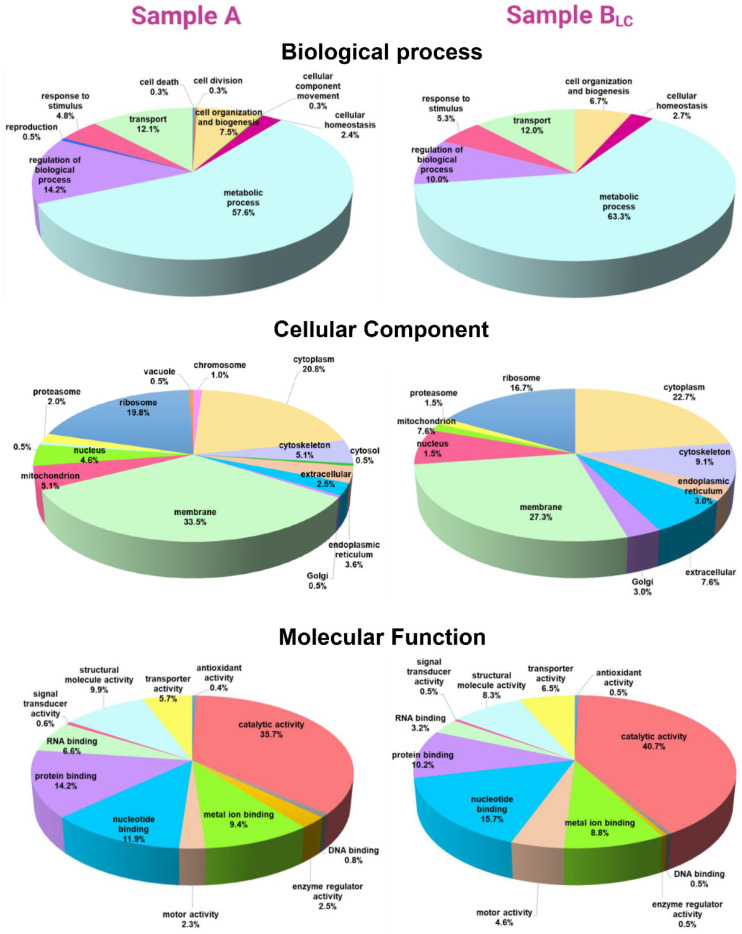
Pie chart of Gene Ontology (GO) distribution terms associated with the identified proteins.

The filtered protein lists were examined for their known GO terms, retrieved by the ProteinCenter application of PD software, and grouped in the respective categories as percentage with respect to the total terms of each sample. The most represented biological process, cellular component, and molecular function were, as expected, similar between sample A and B_LC_ and were linked to metabolic process, membrane, and catalytic activity (Figure 7).

In order to highlight an over-representation of information from GO terms and biological pathways, as well as protein–protein interaction networks, related to our dataset, we converted the two protein lists into orthologous genes of *C. elegans* (Figure 6 and Supplementary material S5), as this model organism is the most investigated one for biomedical research and available in enrichment analysis web applications, contrary to *Anisakis* spp. Orthologous genes are likely conserved through evolution from a common ancestor, may carry out similar function, and are therefore relevant in functional analysis.

**Figure 8:**
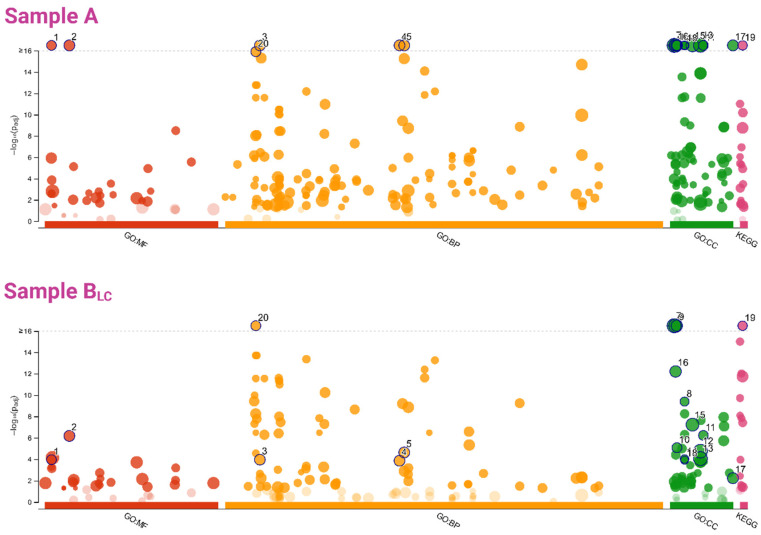
Manhattan plot of GO terms and biological pathways (KEGG) enrichment analysis, results of a multiquery with the two input gene lists (A and B_LC_, obtained from protein). The x-axis represents functional terms that are grouped and color-coded by data sources (e.g., Molecular Function, MF, Biological Process, BP, Cellular Component, CC from GO, and KEGG pathways, are red, green, orange, and violet, respectively). The y-axis shows the adjusted enrichment *p*-values in negative log_10_ scale). Term circles with *p*-values less than 1.0 × 10^−16^ (highly significant) in at least one sample are highlighted and numbered. The circle sizes are in accordance with the corresponding term sizes (i.e., larger terms, which means more hits of the specific term retrieved from the dataset, correspond to larger circles).

Our protein extraction pipeline followed by tandem mass spectrometry analysis led us to a great enrichment of proteins from ribosome and related to carbon metabolism pathway (Figure 8 and Table 2) as well as many other GO terms and biological pathways, such as glycolysis/gluconeogenesis, glyoxylate and dicarboxylate metabolism, metabolic pathways (Supplementary material S6).

**Figure 9:**
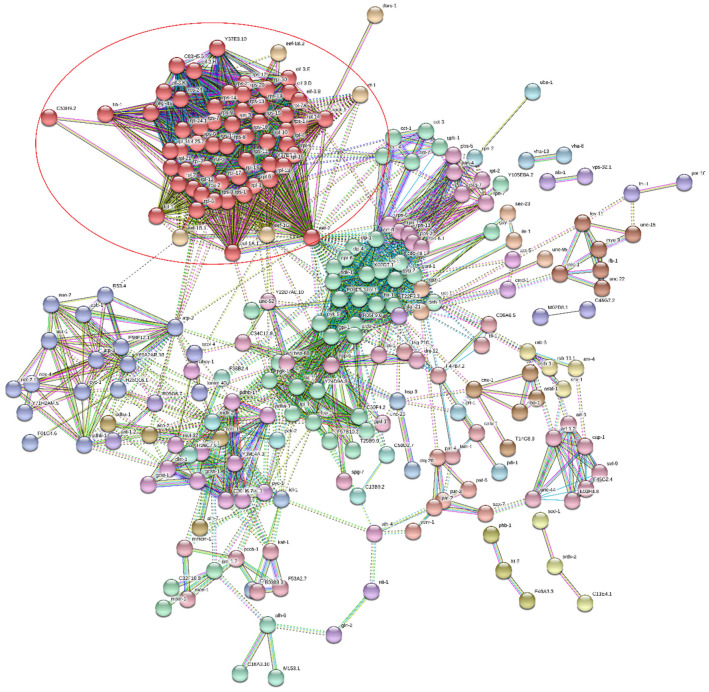
Network analysis of sample A. The network nodes are proteins, and the edges represent the predicted functional associations. The highlighted red cluster is related to ribosomal proteins.

**Figure 10:**
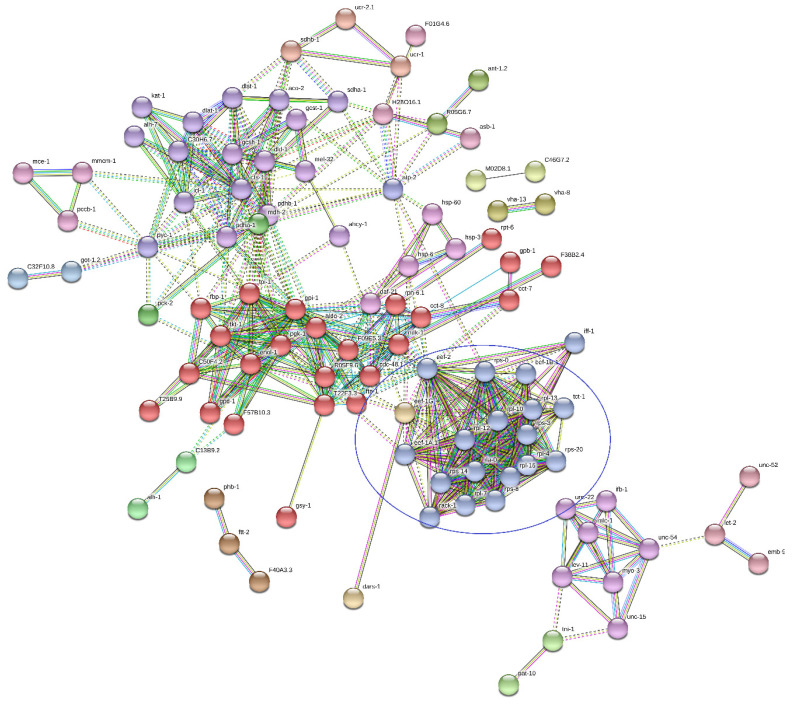
Network analysis of sample B_LC_. The highlighted blue cluster is related to ribosomal proteins.

Consequently, network analysis evidenced a main cluster of ribosomal proteins in sample A (Figure 9) and sample B_LC_ (Figure 10). In fact, a group of proteins at least partially biologically connected present more interactions than a random set of proteins of similar size drawn from the genome; it indicated an enrichment of the ribosome-associated structural constituents and translation.

## 4. Discussion

Clinical laboratories take advantage of the MALDI–TOF MS technology to identify pathogenic microorganisms, including bacteria, yeasts, and filamentous fungi, thanks to the ease of use, speed of analysis, cost-effectiveness, and accuracy of IDs. MALDI–TOF MS analysis allows the detection of different types of biomolecules in a range of concentrations close to sub-femtomoles; in clinical diagnosis, peptides and proteins are fingerprinting classifiers [17]. In particular, one of the main advantages of this particular approach, i.e., rapidity, is grounded on the availability of mass spectra collections unambiguously identifying an organism by matching these spectral libraries with signals obtained from the samples.

Moreover, the applications comply with the European In Vitro Diagnostic Devices Directive (98/79/EC), which means that MALDI–TOF MS is a CE Marking for all in vitro diagnostic (IVD) devices, may be legally commercialized in the EU as a diagnostic tool, and the associated processes are similar to those of medical devices. In fact, the Bruker Biotyper and the VITEK MS system received first CE-IVD status in 2009 and 2011, respectively, and FDA clearance (for both) in 2013 [17]. The technique and the expertise have been growing over time; the implementation of associated software and reference databases, as well as sample preparation kits commercialized by the vendor have allowed researchers and laboratory specialists to both increase the type of microorganisms that can be identified and potentially treat/process “direct samples” (e.g., blood cultures), facilitating the management of infected patients. As an example, Bruker has developed the MBT Sepsityper IVD Kit for the identification of microorganisms from blood cultures using a MALDI–TOF MS platform [18,19,20]. Interestingly, a recent article reported the use of the MBT Sepsityper Kit in order to profile four nematodes (*Dirofilaria repens*, *Dirofilaria immitis*, *Ascaris suum*, and *Ascaris lumbricoides*) [10], which intrigued and prompted us to test this method on *Anisakis* spp. In fact, our future goal, that has driven the preliminary study here presented, is to develop a pipeline for a proteomic profiling of *Anisakis* spp. for diagnostic purposes; the existence of an IVD kit will facilitate this future application of our method.

Although widely used as a diagnostic tool for accurate identification of bacteria, very few studies have tried to translate the advantages of the MALDI–TOF MS technique to clinical parasitology [8,9], and therefore, as far as we know, there are no related commercially MALDI–TOF MS ID spectral library databases.

The aim of our study was to assess the proof of concept that MBT Sepsityper Kit-based protein extraction from *Anisakis* larvae and subsequent MALDI–TOF MS Biotyper-based collection of spectra may provide diagnostic signals of biomedical interest related to this *Anisakis* life stage. The proposed method will work for the identification of already extracted nematode larvae from patients to improve the current diagnostic approaches. Although a variability in the MS peaks was ascertained, a few detected signals were statistically significant in representing all the larvae samples. This evidence is promising for the development of new diagnostic tools for *Anisakis*. We are aware that a definite assessment of the herein hypothesized diagnostic pipeline will require a collection of more *Anisakis* larvae samples, also with different geographical origin, as well as control samples from different nematodes in order to obtain a precise taxonomic typing. Hopefully, in the near future, efforts towards this development will be made in a consortium of research and clinical laboratories.

An interesting outcome of our study was the confirmation that the adopted protein extraction protocol and the MALDI–TOF MS analysis resulted in fingerprinting spectra attributable to peptides and proteins belonging to the plethora of ribosome molecular species, as already known [21]. The molecular weight of ribosomal proteins is within/around the mass range of the linear MALDI–TOF MS acquisition set-up, and our shotgun profiling experiments, by tandem MS on an higher resolution platform, highlighted the enrichment of ribosome proteins in two experimental steps.

Moreover, among the 448 proteins identified in four out of five larvae (Supplementary material S4), we identified also proteins related to infection molecular mechanisms, which have not yet been fully understood [22]. Ingested *Anisakis* has to survive in the highly acidic human stomach, penetrate the gastrointestinal wall, through degradation of the mucosa and submucosa, and migrate to the final location, causing tissue damage and inflammation. At the end, anisakiasis arises from both the tissue damage and the interplay between the host immune system and substances secreted by or contained within the larvae [2]. We identified some harmful antigens (proteinase inhibitor, somatic paramyosin, tropomyosin, and heat shock proteins), including Ani s 2 (UniProtKB accession number: L7V1I9), Ani s 4 (Q14QT4), Ani s 5 (A1IKL2), and Ani s 8 allergens (two isoforms: 1 and 10, A7M6S9 and A1IKL2, respectively), which are present in the Allergome database [23] and registered as *Anisakis* allergenic proteins by WHO/IUIS [24]. Ani s 2 is a cytoskeletal paramyosin protein, showing highly conserved sequences with respect to paramyosins of different origin (other nematodes, insects, shellfish), which account for the cross-reactivity in IgE binding. The excretory–secretory (ES) Ani s 4 allergen is a cysteine-type endopeptidase with inhibitor activity, present both in excretory glands and below the cuticle. Ani s 5 and Ani s 8 are other heat-stable ES allergens and members of the nematode SXP/RAL-2 protein family. In particular, Ani s 5 is assumed to be secreted in the human gastrointestinal tract from the third-stage larvae of *Anisakis simplex*; its putative magnesium ion transporter functional feature may be inferred by its magnesium ion binding capability and structural similarity to Calmodulin [25,26,27]. ES molecules are responsible for a multitude of functions during infection, such as penetration of host tissues and evasion of host immune responses, and at the same time are known to elicit immune responses. Therefore, ES proteins are hypothesized to be the major contributors in clinical manifestation of the disease in humans [28].

The identified Synthase trehalose-6-phosphate (A0A0M3JTQ2) may be important for the infection mechanism because it can be associated with a plethora of physiological and biochemical adaptive mechanisms that parasitic nematodes put in place under adverse environmental conditions, such as the unfavorable pH that *Anisakis* finds in the human gastrointestinal milieu, in order to survive [29].

Proteolytic enzymes, such as peptidases, are responsible for *Anisakis* pathogenicity because of their role in biological pathways linked to fundamental host–pathogen interactions. Among the several peptidases present in our dataset, we identified Metalloendopeptidase (A0A3G5BC99) belonging to the Astacin peptidase family M12A (Pfam code: Pf01400, secreted or membrane-anchored proteases that requires zinc for catalysis) and Carboxypeptidase (A0A158PN74), part of the Peptidase_S10 family (Pf00450). Interestingly, both protein families were recently identified amongst the upregulated transcripts in the pharynx of *A. simplex* and *Anisakis pegreffii* [22]. Moreover, the Metalloendopeptidase mRNA expression level was found to be higher in *A. simplex* third-stage larvae compared to the fourth-stage larvae, enforcing the role of this enzyme as a significant player in host tissues invasion [30].

## 5. Conclusions

A set of MALDI-TOF MS signals was identified as potential consensus “biomarkers” peak list, characterized by specific averaged *m/z* and intensity, for the identification of *Anisakis* spp. from nematode larvae present in patients tissue to improve the current diagnostic approaches. In fact, due to the similarity of symptoms with those of common gastrointestinal disorders and lack of highly skilled microscopists in biomedical laboratories, *Anisakis* infection is often underestimated and alternative diagnostic tools are enviable.

Additionally, the shotgun bottom-up analysis of *Anisakis* proteins, obtained by the extraction method based on the MBT Sepsityper Kit and performed by LC–ESI–MS/MS on a high-resolution platform, underlined the presence, in the nematode extract, of both an enrichment of ribosome proteins and specific proteins potentially associated with molecular mechanisms that accompany infection.

## Figures and Tables

**Table 1:** Selected reference signals for the identification of *Anisakis* spp. DAve, difference between the maximum and the minimum average peak intensities, Ave, peak intensity average, CV, coefficient of variation.

# Peak	*m/z*	DAve	Ave					CV (%)				
			1	2	3	4	5	1	2	3	4	5
1	2597.26	0.97	2.63	2.57	2.55	3.52	3.12	19.32	16.09	15.34	15.86	18.74
2	2654.85	3.6	4.41	7.84	7.78	4.24	5.08	17.66	15.12	12.49	10.57	14.7
3	2704.25	1.26	2.91	3.7	3.51	4.15	2.89	10.86	11.12	15.85	16.27	14.07
4	2724.12	3.66	4.6	7.84	7.4	4.18	4.47	18.86	12.1	12.99	13.48	17.98
5	2733.01	0.98	3.52	4.5	4.26	3.68	3.56	16.22	14.21	9.88	17.98	13.54
6	2741.79	1.25	3.63	4.47	4.47	3.94	3.22	15.67	13.38	11.7	13.73	15.86
7	2758.46	1.13	4.21	3.22	3.98	4.35	3.35	14.8	12.06	8.77	11.37	18.26
8	2781.06	1.35	4.0	4.5	5.11	4.0	3.76	9.79	11.77	9.01	11.98	19.07
9	2807.87	0.82	3.39	3.71	3.61	3.75	2.92	16.69	12.95	11.4	17.88	16.87
10	2827.16	0.6	3.25	3.72	3.55	3.29	3.12	14.97	14.59	14.48	17.82	19.86
11	2834.32	0.71	3.85	4.56	3.99	4.15	4.13	10.02	14.2	14.82	12.6	16.51
12	2840.23	1.8	3.91	2.82	3.98	4.54	4.63	14.68	7.22	11.06	10.73	19.48
13	4969.15	1.4	4.23	4.33	4.24	3.73	2.92	12.47	14.59	12.16	10.67	12.37
14	5335.65	3.25	2.98	4.36	5.38	2.81	6.05	14.79	12.28	12	11.62	15.81
15	5370.57	1.75	2.73	3.57	4.47	3.14	3.91	16.47	16.83	16.68	14.81	12
16	5656.33	7.86	8.48	13.73	12.95	5.87	5.9	10.17	11.46	9.92	14.32	19.42
17	5694.2	1.87	5.67	5.31	5.46	7.19	7.08	7.24	7.8	10.33	18.31	12.79
18	5712.76	6.26	5.97	11.04	9.91	4.78	5.74	8.99	9.07	9.14	13.13	13.56
19	7166.45	5.19	7.98	2.79	3.43	2.98	5.14	14.63	14.19	14.1	14.43	15.45

**Table 2:** GO terms and biological pathways (KEGG) enrichment analysis.

Term Group	Circle Number (Figure 8)	Term Name	Term id	Adjusted *p*-Value	
				Sample A	Sample B_LC_
MF	1	structural constituent of ribosome	GO:0003735	4.07 × 10^−26^	1.10 × 10^−04^
	2	structural molecule activity	GO:0005198	1.42 × 10^−18^	6.53 × 10^−07^
BP	3	translation	GO:0006412	5.30 × 10^−21^	1.10 × 10^−04^
	4	peptide biosynthetic process	GO:0043043	1.11 × 10^−20^	1.38 × 10^−04^
	5	amide biosynthetic process	GO:0043604	2.53 × 10^−20^	2.32 × 10^−05^
	20	generation of precursor metabolites and energy	GO:0006091	1.23 × 10^−16^	6.24 × 10^−18^
CC	6	cytoplasm	GO:0005737	2.19 × 10^−82^	6.58 × 10^−40^
	7	intracellular	GO:0005622	7.43 × 10^−55^	1.82 × 10^−22^
	8	cytosolic ribosome	GO:0022626	7.86 × 10^−38^	4.04 × 10^−10^
	9	cytosol	GO:0005829	3.35 × 10^−36^	1.37 × 10^−21^
	10	ribosome	GO:0005840	4.02 × 10^−28^	8.32 × 10^−06^
	11	ribosomal subunit	GO:0044391	1.05 × 10^−27^	5.91 × 10^−07^
	12	intracellular organelle	GO:0043229	7.65 × 10^−23^	1.76 × 10^−05^
	13	organelle	GO:0043226	1.01 × 10^−21^	8.60 × 10^−05^
	14	cytosolic large ribosomal subunit	GO:0022625	1.98 × 10^−20^	9.33 × 10^−05^
	15	protein-containing complex	GO:0032991	6.21 × 10^−20^	5.83 × 10^−08^
	16	mitochondrion	GO:0005739	3.42 × 10^−19^	6.19 × 10^−13^
	17	ribonucleoprotein complex	GO:1990904	6.95 × 10^−18^	5.88 × 10^−03^
	18	cytosolic small ribosomal subunit	GO:0022627	4.34 × 10^−17^	1.23 × 10^−04^
KEGG	19	carbon metabolism	KEGG:01200	9.55 × 10^−20^	1.69 × 10^−27^

## Supplementary Materials

The following are available online at https://www.mdpi.com/2073-4425/11/6/693/s1, Supplementary material S1: List of identified proteins by LC–ESI–MS/MS; Supplementary material S2: List of identified proteins by LC–ESI–MS/MS and belonging to Rhabditida taxonomy order; Supplementary material S3: List of Rhabditida proteins identified in at least four out of five larvae of samples A and B_LC_, Supplementary material S4: List of Rhabditida proteins identified in at least four out of five larvae of samples A and B_LC_ and filtered by collapsing proteins with the same name into one single hit and deleting unknown proteins; Supplementary material S5: Results of gene orthology conversion; Supplementary material S6: Functional analysis enrichment.

## References

[1] Nieuwenhuizen N.E., Lopata A.L. (2014). Allergic Reactions to Anisakis Found in Fish. Curr. Allergy Asthma Rep..

[2] Nieuwenhuizen N.E. (2016). *Anisakis*—Immunology of a foodborne parasitosis. Parasite Immunol..

[3] Hochberg N.S., Hamer D.H. (2010). Anisakidosis: Perils of the Deep. Clin. Infect. Dis..

[4] Putignani L., Del Chierico F., Onori M., Mancinelli L., Argentieri M., Bernaschi P., Coltella L., Lucignano B., Pansani L., Ranno S. (2011). MALDI-TOF mass spectrometry proteomic phenotyping of clinically relevant fungi. Mol. Biosyst..

[5] Del Chierico F., Masotti A., Onori M., Fiscarelli E., Mancinelli L., Ricciotti G., Alghisi F., Dimiziani L., Manetti C., Urbani A. (2012). MALDI-TOF MS proteomic phenotyping of filamentous and other fungi from clinical origin. J. Proteom..

[6] Del Chierico F., Petrucca A., Vernocchi P., Bracaglia G., Fiscarelli E., Bernaschi P., Muraca M., Urbani A., Putignani L. (2014). Proteomics boosts translational and clinical microbiology. J. Proteom..

[7] Greco V., Piras C., Pieroni L., Ronci M., Putignani L., Roncada P., Urbani A. (2018). Applications of MALDI-TOF mass spectrometry in clinical proteomics. Expert Rev. Proteom..

[8] Murugaiyan J., Roesler U. (2017). MALDI-TOF MS Profiling-Advances in Species Identification of Pests, Parasites, and Vectors. Front. Cell. Infect. Microbiol..

[9] Feucherolles M., Poppert S., Utzinger J., Becker S.L. (2019). MALDI-TOF mass spectrometry as a diagnostic tool in human and veterinary helminthology: A systematic review. Parasites Vectors.

[10] Nagorny S.A., Aleshukina A.V., Aleshukina I.S., Ermakova L.A., Pshenichnaya N.Y. (2019). The application of proteomic methods (MALDI-toff MS) for studying protein profiles of some nematodes (dirofilaria and ascaris) for differentiating species. Int. J. Infect. Dis..

[11] Distler U., Kuharev J., Navarro P., Tenzer S. (2016). Label-free quantification in ion mobility-enhanced data-independent acquisition proteomics. Nat. Protoc..

[12] g:Profiler. https://biit.cs.ut.ee/gprofiler/orth.

[13] Reimand J., Arak T., Adler P., Kolberg L., Reisberg S., Peterson H., Vilo J. (2016). g:Profiler—A web server for functional interpretation of gene lists (2016 update). Nucleic Acids Res..

[14] STRING. https://string-db.org/.

[15] Szklarczyk D., Gable A.L., Lyon D., Junge A., Wyder S., Huerta-Cepas J., Simonovic M., Doncheva N.T., Morris J.H., Bork P. (2019). STRING v11: Protein–protein association networks with increased coverage, supporting functional discovery in genome-wide experimental datasets. Nucleic Acids Res..

[16] Stryiński R., Mateos J., Pascual S., González Á.F., Gallardo J.M., Łopieńska-Biernat E., Medina I., Carrera M. (2019). Proteome profiling of L3 and L4 Anisakis simplex development stages by TMT-based quantitative proteomics. J. Proteom..

[17] Holmes D.T., Romney M.G., Angel P., DeMarco M.L. (2020). Proteomic Applications in Pathology and Laboratory Medicine: Present State and Future Prospects. Clin. Biochem..

[18] Jeddi F., Yapo-Kouadio G.C., Normand A.-C., Cassagne C., Marty P., Piarroux R. (2017). Performance assessment of two lysis methods for direct identification of yeasts from clinical blood cultures using MALDI-TOF mass spectrometry. Med. Myco..

[19] Kayin M., Mert B., Aydemir S., Özenci V. (2019). Comparison of rapid BACpro® II, Sepsityper® kit and in-house preparation methods for direct identification of bacteria from blood cultures by MALDI-TOF MS with and without Sepsityper® module analysis. Eur. J. Clin. Microbiol. Infect. Dis..

[20] Luethy P.M., Johnson J.K. (2019). The Use of Matrix-Assisted Laser Desorption/Ionization Time-of-Flight Mass Spectrometry (MALDI-TOF MS) for the Identification of Pathogens Causing Sepsis. J. Appl. Lab. Med..

[21] Ryzhov V., Fenselau C. (2001). Characterization of the protein subset desorbed by MALDI from whole bacterial cells. Anal. Chem..

[22] Cavallero S., Lombardo F., Su X., Salvemini M., Cantacessi C., D’Amelio S. (2018). Tissue-specific transcriptomes of Anisakis simplex (sensu stricto) and Anisakis pegreffii reveal potential molecular mechanisms involved in pathogenicity. Parasites Vectors.

[23] Mari A., Rasi C., Palazzo P., Scala E. (2009). Allergen databases: Current status and perspectives. Curr. Allergy Asthma Rep..

[24] Aibinu I.E., Smooker P.M., Lopata A.L. (2019). Anisakis Nematodes in Fish and Shellfish- from infection to allergies. Int. J. Parasitol. Parasites Wildl..

[25] Audicana M.T., Kennedy M.W. (2008). Anisakis simplex: From Obscure Infectious Worm to Inducer of Immune Hypersensitivity. CMR.

[26] Kobayashi Y., Ishizaki S., Shimakura K., Nagashima Y., Shiomi K. (2007). Molecular cloning and expression of two new allergens from Anisakis simplex. Parasitol. Res..

[27] García-Mayoral M.F., Treviño M.A., Pérez-Piñar T., Caballero M.L., Knaute T., Umpierrez A., Bruix M., Rodríguez-Pérez R. (2014). Relationships between IgE/IgG4 Epitopes, Structure and Function in Anisakis simplex Ani s 5, a Member of the SXP/RAL-2 Protein Family. PLoS Negl. Trop. Dis..

[28] Mehrdana F., Buchmann K. (2017). Excretory/secretory products of anisakid nematodes: Biological and pathological roles. Acta Vet. Scand..

[29] Łopieńska-Biernat E., Stryiński R., Dmitryjuk M., Wasilewska B. (2019). Infective larvae of Anisakis simplex (Nematoda) accumulate trehalose and glycogen in response to starvation and temperature stress. Biol. Open.

[30] Kim J.-H., Kim J.-O., Jeon C.-H., Nam U.-H., Subramaniyam S., Yoo S.-I., Park J.-H. (2018). Comparative transcriptome analyses of the third and fourth stage larvae of Anisakis simplex (Nematoda: Anisakidae). Mol. Biochem. Parasitol..

